# One-Pot Sr-Free LaFeO_3_/CeO_2_-Based Electrocatalytic Composites: Effect of Cerium and Lanthanum Interplay Between Perovskite and Fluorite

**DOI:** 10.3390/ma19112361

**Published:** 2026-06-02

**Authors:** Laura Valentino, Francesca Deganello, Leonarda Francesca Liotta, Giuseppe Marcì, Chiara Aliotta

**Affiliations:** 1 National Research Council of Italy (CNR), Institute of Nanostructured Materials (ISMN) Palermo, Via Ugo La Malfa 153, 90146 Palermo, Italy; lauravalentino@cnr.it (L.V.); francesca.deganello@cnr.it (F.D.); leonardafrancesca.liotta@cnr.it (L.F.L.); 2 Department of Engineering, University of Palermo, Viale delle Scienze, Building 6, 90128 Palermo, Italy; giuseppe.marci@unipa.it

**Keywords:** perovskite oxides, doped lanthanum ferrite, perovskite–ceria composites, one-pot solution combustion synthesis, oxygen vacancies, defects engineering, Raman spectroscopy, reduction properties, oxygen reduction reaction, solid oxide fuel cells

## Abstract

**Highlights:**

**What are the main findings?**

- SCS enables rapid one-pot synthesis of perovskite-fluorite based composites.
- Perovskite lattice expands due to A- and B-site defect chemistry changes.
- Raman reveals defect-induced distortion of BO_6_ octahedra.

**What are the implications of the main findings?**

- Perovskite growth on pre-formed CeO_2_ induces a defect-rich structure.
- One-pot synthesis reduces the amount of ceria in the composite.
- Suitable design strategy for SOFC cathodes and redox-active materials.

**Abstract:**

Perovskite-type oxides are among the most promising cathodes for intermediate-temperature solid oxide fuel cells (IT-SOFCs) due to their mixed ionic–electronic conductivity and compositional flexibility. Many high-performance cathodes rely on Sr substitution at the A-site, often associated with surface segregation and long-term degradation. In this work, we explore an alternative strategy based on defect engineering and phase interactions in Sr-free composites. Perovskite-fluorite composites based on LaFe_0.8_Co_0.2_O_3_ were synthesized through a one-pot route designed to promote the formation of a perovskite phase and a limited amount of fluorite-type ceria. This approach allows the introduction of small fractions of Ce into the perovskite lattice, favoring the cooperative coexistence with La-doped CeO_2_. Structural, microstructural and spectroscopic characterization indicates that Ce influences the crystallization pathway and composite defect chemistry. Variations in lattice parameters and Raman features suggest modifications of perovskite structure consistent with defect formation and lattice distortion. Reduction properties and electrical conductivity measurements indicate that Ce incorporation in the perovskite and oxide interaction affect charge transport and oxygen mobility. The electrochemical results demonstrate that the optimal trade-off between activation energy (Ea) and polarization resistance (Rp) is achieved for the sample, with a nominal cerium content, Ce/(La + Ce) of 0.16. Moreover, the electrochemical properties are found to correlate with the nominal cerium content, which regulates defect chemistry and the resulting composite composition. Overall, results suggest that the one-pot synthesis promotes beneficial interactions between the perovskite and ceria phases, allowing the development of Sr-free ferrite-based materials with enhanced functional properties, minimizing the amount of ceria in the composite.

## 1. Introduction

Solid oxide fuel cells (SOFCs) are widely regarded as one of the most promising technologies for sustainable energy conversion, offering high electrical efficiency and fuel flexibility while minimizing environmental impact. By operating outside the traditional carbon-based energy chain, SOFCs can efficiently utilize hydrogen and other low-carbon fuels, contributing to the reduction of greenhouse gas emissions and supporting the transition toward sustainable energy systems [1]. Intermediate-temperature SOFCs (IT-SOFCs), operating in the range of 500–800 °C, have attracted increasing attention because they allow the use of less-expensive materials and improved long-term durability compared with conventional high-temperature systems. Despite these advantages, the cathodic oxygen reduction reaction (ORR) remains the major kinetic bottleneck limiting the overall performance of SOFC devices. The ORR involves a sequence of elementary steps including oxygen adsorption, oxygen dissociation, electron transfer, and incorporation of oxygen ions into the oxide lattice of the cathode and electrolyte materials through oxygen vacancies. Once incorporated, oxygen ions migrate through the lattice via vacancy hopping mechanisms, while electronic transport proceeds through small-polaron hopping processes governed by the position of the Fermi level. Consequently, high-performance cathode materials must exhibit mixed ionic–electronic conductivity (MIEC), in which the concentration and mobility of oxygen vacancies and electronic carriers play a fundamental role in determining catalytic activity. Among the various MIEC materials reported in the literature, perovskite-type oxides with the general formula ABO_3_ have attracted considerable interest because of their compositional flexibility and tunable defect chemistry. Aliovalent substitutions at both the A and B lattice sites allow the control of structural distortions, oxygen vacancy concentration, and electronic structure, thereby enabling the optimization of catalytic and transport properties [2]. Lanthanum ferrite (LaFeO_3_)-based perovskites represent promising cathode materials due to their good chemical stability and the abundance of iron, which is not considered a critical raw material [3]. The development of cathode materials based on abundant elements is becoming increasingly important in the context of growing global competition for strategic raw materials. To enhance the electrochemical performance of LaFeO_3_, several doping strategies have been explored. Partial substitution of Fe with transition metals such as Co is known to enhance electronic conductivity and oxygen exchange kinetics by modifying the metal–oxygen bond covalency and the electronic structure of the oxide lattice [4,5]. At the same time, A-site substitution has been widely used to tune the defect chemistry of perovskite oxides and increase the concentration of oxygen vacancies. Many high-performance SOFC cathodes rely on Sr substitution at the A-site, as in La_1−x_Sr_x_Co_1−y_Fe_y_O_3−δ_ materials [6,7,8]. However, the use of Sr is associated with well-known stability issues, including surface segregation and the formation of insulating secondary phases during operation, which can lead to long-term degradation of the electrode performance [9]. For this reason, increasing attention has recently been devoted to alternative strategies capable of improving the catalytic properties of ferrite perovskites while avoiding Sr substitution [10,11]. One promising approach consists of exploiting defect engineering strategies based on the introduction of redox-active dopants capable of modifying the defect chemistry of the perovskite lattice [12,13,14,15,16]. In particular, small amounts of cerium introduced at the A-site can influence charge compensation mechanisms and promote the formation of oxygen vacancies while modifying the oxidation state distribution of the B-site transition metals [15,16]. The presence of Ce can also introduce additional redox flexibility due to the Ce^4+^/Ce^3+^ couple, which may influence oxygen exchange and catalytic processes [12,17,18,19]. However, according to the literature, Ce exhibits a limited solubility, typically below 10 mol% on Ce-doped LaFeO_3_ and related systems. For example, Kim et al. reported that, via the EDTA-citrate complexing method, Ce 5 mol% is the maximum limit in LaFeO_3_, and the attempt to dope with 7% leads to the formation of CeO_2_ [12]. Similar results were obtained for Ce-doped LaFeO_3_ nanofibers produced by electrospinning, in which case the solubility limit was 10 mol% [20]. To the best of our knowledge, no articles have reported on Ce-doped LaFe_0.8_Co_0.2_O_3_, although several authors have studied the effects of cerium doping on LaFeO_3_, pointing to cerium’s limited solubility. Looking at other La-based perovskites, a limited accommodation of cerium in the perovskite lattice, as cobaltite (LaCoO_3_), was also demonstrated [21].

The role of defects engineering in controlling the functional properties of perovskite oxides has been widely recognized as a powerful strategy to tune catalytic activity and charge transport properties [22]. Moreover, defect formation, such as oxygen vacancy generation, is also a key factor in determining the catalytic performance of perovskite oxides in oxidation reactions [23]. Besides compositional tuning, another important strategy to enhance cathode performance consists of combining perovskite MIEC materials with oxy gen-ion conducting oxides such as cerium oxide (CeO_2_). Doped ceria is widely used because of its high oxygen ion conductivity and oxygen storage capability. This behavior is also related to its fluorite-type crystal structure, which can accommodate aliovalent cations with relatively large ionic radii, promoting the formation of oxygen vacancies and thereby enhancing ionic transport [24]. As a result, perovskite-fluorite composite electrodes have been extensively investigated for both electrochemical and catalytic applications [5,13,25,26]. The high catalytic activity of ceria is closely related to its ability to form and rearrange oxygen vacancies, which can dynamically adapt under reaction conditions and strongly influence catalytic processes. However, conventional composite cathodes are typically prepared by mechanical mixing of the two phases, and relatively large amounts of ceria—often up to 30–40 wt.%—are required to achieve sufficient ionic conductivity. Such approaches may limit the potential cooperative effects between the perovskite and ceria phases [27]. In recent years, attention has been devoted to synthesis strategies that allow the simultaneous formation of the different oxide phases in a single preparation step [15,16,28]. One-pot synthesis approaches can influence the crystallization pathway of the materials and promote interactions between the phases during their formation. These synthesis induced interactions may affect the defect chemistry, redox properties, and catalytic behavior of the resulting composite materials [29,30]. Similar phase interactions have been shown to influence charge transfer processes and catalytic activity in oxide systems involving ceria and transition metal oxides. For example, the interfacial interaction, arising from one pot synthesis of a nanocomposite containing a major cubic perovskite-doped BaCoO_3_ phase and a minor hexagonal swedenborgite phase, notably improves the electrocatalytic performance of OER [16]. Moreover, lattice oxygen mobility is enhanced by the mutual synergy between double and single layered perovskites [15]. Similar phase interactions in oxide systems containing ceria and transition metal oxides have been shown to influence charge transfer processes and catalytic activity [5,31]. In this context, the possibility of exploiting the interactions occurring during a one-pot synthesis to design perovskite–ceria composites with reduced ceria content remains relatively unexplored. At the same time, developing ferrite-based cathodes that avoid Sr substitution while maintaining high catalytic activity is an important objective in the perspective of improving long-term stability [32,33].

In the present work, (La,Ce)Fe_0.8_Co_0.2_O_3_-(La,Ce)O_2_ composites are purposely designed to promote the formation of a perovskite-fluorite composite during one-pot solution combustion synthesis (SCS). The novelty of this approach lies in the exploitation of the low solubility of Ce in the perovskite lattice, promoting a cooperative coexistence between Ce-doped LaFe_0.8_Co_0.2_O_3_ and La-doped CeO_2_. The interplay of Ce and La between the perovskite and fluorite structure contributes to defect formation in both phases, while the favorable interaction of the perovskite with CeO_2_ influences the reduction behavior, oxygen exchange properties and electrochemical properties of the system. The research goal is to develop novel Sr-free perovskite-fluorite composites with improved functional properties while minimizing the amount of ceria in the composite.

## 2. Materials and Methods

### 2.1. Composite Preparation

Powders with nominal compositions La_1.05_Ce_0.2_Fe_0.8_Co_0.2_O_3_, LaCe_0.2_Fe_0.8_Co_0.2_O_3_, La_0.8_Ce_0.2_Fe_0.8_Co_0.2_O_3_, La_0.9_Ce_0.1_Fe_0.8_Co_0.2_O_3_, and La_0.95_Ce_0.05_Fe_0.8_Co_0.2_O_3_ were synthesized via the solution combustion method using La(NO_3_)_3_·6H_2_O (>99.0%, Fluka Analytical, Chemie, Steiheim, Germany), Ce(NO_3_)_3_·6H_2_O (99.99%, Aldrich, Sigma-Aldrich Chemie, Steinheim, Germany), Fe(NO_3_)_3_·9H_2_O (≥99.95%, Sigma-Aldrich, St. Louis, MO, USA), and Co(NO_3_)_3_·6H_2_O (≥99.99%, Fluka, Sigma-Aldrich Chemie, Steinheim, Germany) as metal precursors and anhydrous citric acid (≥99.5%, Aldrich, Sigma-Aldrich Chemie, Steinheim, Germany) employed as the fuel. The combustion parameter was set to a reducer-to-oxidizer ratio (φ) of 1.5 with a fuel-to-metal cation molar ratio of 2. The pH was adjusted to 6 using an ammonia solution (28.0–30.0% *v*/*v*, Aldrich, Sigma-Aldrich Chemie, Steinheim, Germany). Ammonium nitrate (NH_4_NO_3_, 98.7%, GPR Rectapur, Leuven, Belgium) was added as an additional oxidant. The resulting aqueous solution was magnetically stirred at 80 °C on a hotplate (VWR Advanced, VMS-C7, Darmstadt, Germany) in a 1 L stainless-steel beaker until a homogeneous gel was formed. The temperature of the hotplate was then set to 350 °C, initiating a self-sustaining combustion reaction that, after a few seconds, produced a brown powder in the case of La_1.05_Ce_0.2_Fe_0.8_Co_0.2_O_3_ and LaCe_0.2_Fe_0.8_Co_0.2_O_3_ and a dark-brown powder for the samples La_0.8_Ce_0.2_Fe_0.8_Co_0.2_O_3_, La_0.9_Ce_0.1_Fe_0.8_Co_0.2_O_3_ and La_0.95_Ce_0.05_Fe_0.8_Co_0.2_O_3_. The as-burned powders were subsequently calcined in static air at 1000 °C. The names of the prepared samples, their nominal composition, and their nominal Ce content, in terms of Ce/(La + Ce), are reported in Table 1. The samples are labeled according to their nominal La and Ce content in the A-site of the perovskite formula, with a fixed B-site composition (Fe 80 mol%, Co 20 mol%). The first number in the label corresponds to the La mol%, whereas the second one indicates the Ce mol%. This nomenclature is adopted to clearly highlight the variation in A-site composition among the prepared materials.

### 2.2. Membrane Electrode Assemblies

Membrane electrode assemblies (MEAs) with a symmetric cell configuration were prepared to perform electrochemical impedance spectroscopy characterization. The electrolyte powder, Ce_0.8_Sm_0.2_O_2−x_ (SDC), was synthesized using a solution combustion reported in a previous paper [24]. Full-density SDC pellets were obtained from the calcined powder at 500 °C by pressing isostatically at 3.4 ton/cm^2^ and by sintering in static air at 1250 °C for 10 h. The resulting pellets had a diameter of approximately 10 mm and a thickness of about 1 mm. For the cathode deposition, the powders were ground with ethanol (99.8%, Sigma-Aldrich) and Polyethylene Glycol 400 (PEG-400, Sigma-Aldrich) and then mixed for 30 min in an ultrasonic bath to obtain a homogeneous suspension. The resulting inks were applied by screen-printing on both sides of the sintered pellets, dried at 200 °C for 1 h *per* side, and finally heat-treated at 1100 °C for 2 h in static air.

### 2.3. Chemical-Physical and Electrochemical Characterization

Powder XRD analysis was performed using a Rigaku MiniFlex600 powder diffractometer (Rigaku Europe SE, Neu-Isenburg, Germany) with Cu Ka radiation (40 kV, 15 mA), Ni filtration, X-ray fluorescence reduction, a 0.05° step size and 1°/min speed. Rietveld refinement of the diffraction patterns was carried out using the GSAS-II package version 5365 [34], refining the Chebyschev polynomial background profile, lattice constants, atomic coordinates, scale factors and crystal size parameters. Database PDF-5+ 2025 (database version 4.2502, software version 4.25.0.3) released by ICDD (International Centre for Diffraction Data, USA) [35] was used for qualitative structural analysis and for the Crystallographic Information Files (cif) files for the refinement.

Raman spectra were recorded using a DXR3 Raman Microscope (Thermo Fisher Scientific S.p.A., MI, Italy) equipped with a 532 nm laser source and characterized by a spatial and confocal depth resolution of 1 and 2 μm, respectively. Spectral processing and analysis were performed using OMNIC for Dispersive Raman software released by Thermo Fisher Scientific Inc. (Thermo Fisher Scientific S.p.A., MI, Italy) For each sample, measurements were collected with the laser power set to 1 mW at 6 different points on the surface, and the resulting spectra were averaged to obtain a representative profile.

Hydrogen temperature programmed reduction (TPR-H_2_) measurements were carried out with a Micromeritics AutoChem 2910 (Micromeritics Instrument Corp., Norcross, GA, USA). Automated Catalyst Characterization System, equipped with a thermal conductivity detector (TCD). About 0.1 g of sample was used for each measurement. The samples were pre-treated with 5 vol% O_2_/He at 30 mL/min, heating up (10 °C/min) to 300 °C and holding at this temperature for 30 min. After returning to room temperature, the gas mixture of 5 vol% H_2_ in Ar was introduced at 30 mL/min into the sample tube. During the analysis, the temperature increased up to 900 °C at a rate of 10 °C/min. The effluent gas was analyzed with a TCD.

Scanning electron microscopy (SEM) was performed using a FEI Quanta 200 ESEM microscope (Thermo-Scientific, Waltham, MA, USA), operating at 30 kV. Before being subjected to SEM analysis, the samples were gilded with a thin layer of gold. On the other hand, an electron microprobe used in an energy dispersive mode (EDX) was employed to obtain information on the actual metal-content percentage present in the samples.

**Figure A1:**
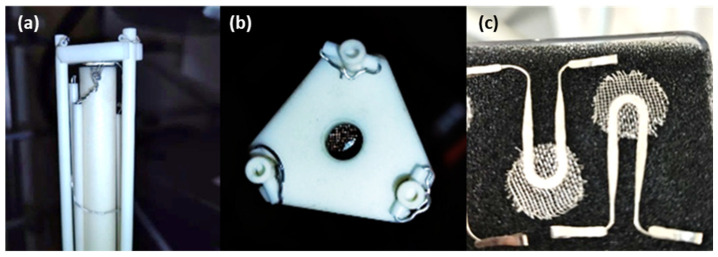
A detailed view of the electrochemical cell setup: (**a**) front-view photograph showing the half-cell configuration; (**b**) top-view photograph of the setup; and (**c**) Pt electrode meshes.

Impedance spectroscopy measurements were conducted employing a Methrohm Autolab impedance analyzer (Metrohm Italian S.r.l., VA) (AC voltage of 0.3 V) equipped with a ProboStat (NorECs AS, Sandvika, Norway) in the 1 × 10^5^–1 × 10^−2^ Hz range between 700–850 °C (50 °C steps, with 1 h equilibration at each temperature) in air. The electrochemical cell setup employs two Pt electrode meshes as current collectors in direct contact with both electrodes, applying a constant mechanical pressure on the half-cell through a spring-loaded system (Figure A1). The impedance data were analyzed with ZView software version 4.0, released by Scribner (USA).

## 3. Results and Discussion

The perovskite-fluorite-based composites (Table 1) were synthesized via solution combustion synthesis (SCS). This method was selected due to its simplicity, rapid reaction kinetics, and cost-effectiveness. Moreover, SCS allows selecting several processing parameters to control the microstructural and redox properties of the final powder [36,37].

**Figure 1:**
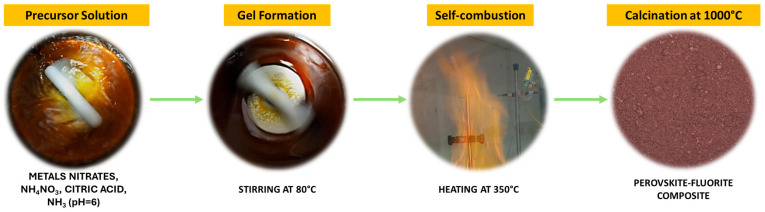
Schematic illustration of solution combustion synthesis used to prepare the perovskite-fluorite composite. The pictures refer to La105Ce20 as a representative sample. Step 1: formation of the precursor solution; step 2: formation of the gel network; step 3: self-combustion resulting in the as-burned powder; step 4: thermal treatment.

The overall synthesis procedure is schematically illustrated in Figure 1, referring to La105Ce20 as a representative sample. The initial solution was formed by all the reagents and additives dissolved in the same beaker (step 1). The formation of the gel, obtained after solvent evaporation and complexation reactions between citric acid and metal cations (step 2), allows interconnections between the various components, fixing them in a network that, through the following self-combustion (step 3), has a direct influence on the final powder phase composition and microstructure, as reported in the literature [37]. The as-burned powder was formed within a few seconds, and this rapid process promotes the simultaneous reaction of all precursors, enabling a homogeneous distribution and a fast crystallization of the perovskite- and fluorite-based phases directly from the gel network. A subsequent thermal treatment at 1000 °C was necessary to stabilize the final perovskite-based composites and eliminate any residual carbon (step 4).

**Figure 2:**
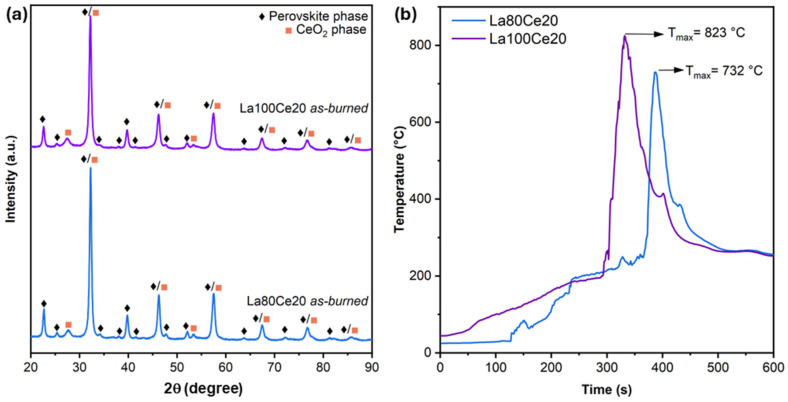
XRD pattern diffraction (**a**) and the Temperature/Time profiles measured during combustion (**b**) of the as-burned powders, La80Ce20 and La100Ce20.

Figure 2a shows the XRD diffraction pattern of the as-burned La100Ce20 and La80Ce20 powders, as examples, whereas Figure 2b contains the relative Temperature vs. Time profiles measured during the self-combustions. In the as-burned powders, the main oxide phases, namely the perovskite phase and fluorite-type ceria phase, were already formed (Figure 2a). Temperature–time profiles indicate that the self-combustion process is completed within 100–150 s, with a maximum temperature of 750–800 °C (Figure 2b). The small shoulder observed on the right side of each peak suggests that phase formation proceeds through successive combustion steps (Figure 2b), in agreement with the multiphase nature of the obtained material.

**Figure 3:**
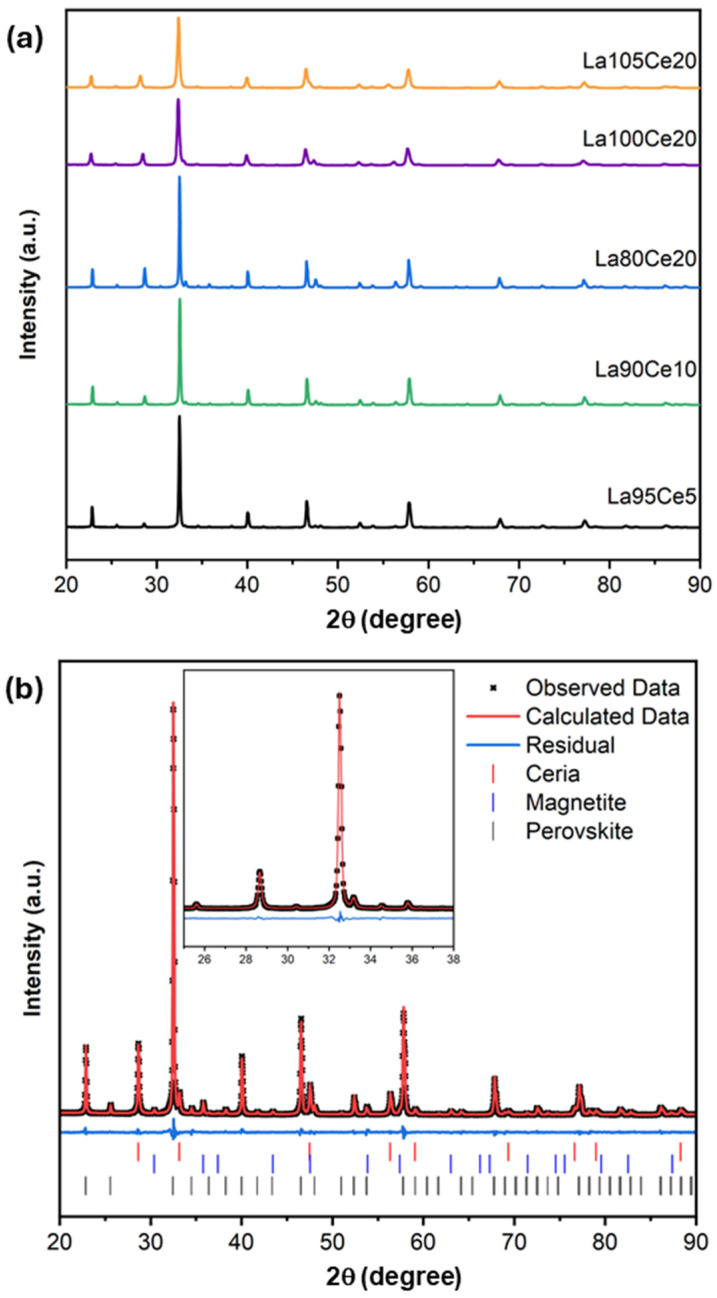
XRD patterns of the investigated perovskite-fluorite composites (**a**) and Rietveld refinement of La80Ce20 with the phase 2θ positions of ICCD PDF Cards (**b**).

X-ray diffraction analysis coupled with Rietveld refinement was performed on the calcined powders. Figure 3a shows the XRD patterns of the investigated samples, Figure 3b shows the corresponding Rietveld refinement for La80Ce20, as a representative example, whereas the refined structural parameters are summarized in Table 2 and Table A1, including the relative standard deviation values. As a general comment, the main peak of the perovskite phase at about ~32.4 °2θ is visible in all the patterns, together with the main peak of the ceria phase at about ~28.6 °2θ. In detail, according to the synthesis design, the samples La105Ce20 and La100Ce20 exhibit a biphasic system consisting of a perovskite-type oxide with orthorhombic symmetry (space group Pbnm, ICDD PDF Card—01-082-9771) and CeO_2_ fluorite-type oxide (space group Fm-3m, ICDD PDF Card—00-004-0593). Instead, the samples La80Ce20, La90Ce10 and La95C5 contain three phases, with magnetite, Fe_3_O_4_ (space group Fd-3m, ICDD PDF Card—01-080-7683), appearing as the third phase. In all specimens, the perovskite phase is the predominant one, whereas the amount of the fluorite phase increases with increasing cerium content.

As shown in Table 2, perovskite phases exhibit unit cell volumes in the 238.58–239.51 Å^3^ range, evidencing an expansion if compared to the value of 237.95 Å^3^ for LaFe_0.8_Co_0.2_O_3_ (ICDD PDF Card—01-082-9771). This expansion could be explained by a partial incorporation of cerium in the perovskite structure, although Ce^4+^ and Ce^3+^ are expected to contract the perovskite volume since they have a smaller ionic radius (i.r. 1.14 Å and 1.34 Å in 12th-fold, respectively) than La^3+^ (i.r. 1.36 in Å 12th-fold). In fact, another structural effect prevails in this case, that is cerium accommodation in the A-site probably forces a Fe^3+^ (i.r. 0.645 Å in 6th-fold) reduction to Fe^2+^ (i.r. 0.78 Å in 6th-fold) for maintaining the charge balance in the system, causing a cell volume expansion as highlighted in the literature [12,18,39,40,41,42]. However, the observed cell expansion cannot be justified by the incorporation of cerium at the A-site of the perovskite, since Ce exhibits a limited and synthesis-dependent solubility, typically below 10 mol% according to the literature on Ce-doped LaFeO_3_ and related systems [12,20,21]. The low cerium solubility is further confirmed by the phase composition results in Table 2, which indicate that the ceria phase is already detected in the La95Ce5 sample, and thus the solubility of cerium in the perovskite structure is below 5 mol%. Moreover, as the nominal cerium content increases in the La95Ce5, La90Ce10, and La80Ce20 sample series, the fraction of ceria as a separate phase progressively grows, in agreement with the consideration that only a small amount of cerium is incorporated into the perovskite lattice. Despite this limited solubility, a systematic increase in unit cell volume is observed from La95Ce5 to La80Ce20 (Table 2). Since cerium incorporation into the perovskite is minimal and almost equal in all the samples, this volume expansion cannot be attributed directly to Ce substitution. Thus, it is likely attributed to a progressive increase in Fe^2+^ content, due to the reduction of Fe^3+^, along with a partial reduction of Co^3+^ (i.r. 0.545 Å, in 6th-fold) to Co^2+^ (i.r. 0.745 Å, in 6th-fold) within the perovskite structure, resulting in lattice expansion [12,18,39,40,41,42,43]. Furthermore, a possible cause of this increased defectivity could arise from an interaction between the forming perovskite phase and ceria during the one-pot synthesis, where the precursors of the perovskite phase are in contact with ceria prior to crystallization. In fact, the formation of cerium oxide is thermodynamically favored over perovskite during combustion synthesis steps and allows crystal growth of the perovskite phase on the already formed CeO_2_-based powder.

A further insight is provided by the La100Ce20 and La105Ce20 samples which, despite quite similar nominal cerium contents (Table 1), exhibit perovskite unit cell volumes lower than that of La80Ce20 (Table 2). This can be attributed to a reduced degree of lattice expansion, likely due to a lower amount of reduced B-site cations (Fe^2+^/Co^2+^).

**Figure A2:**
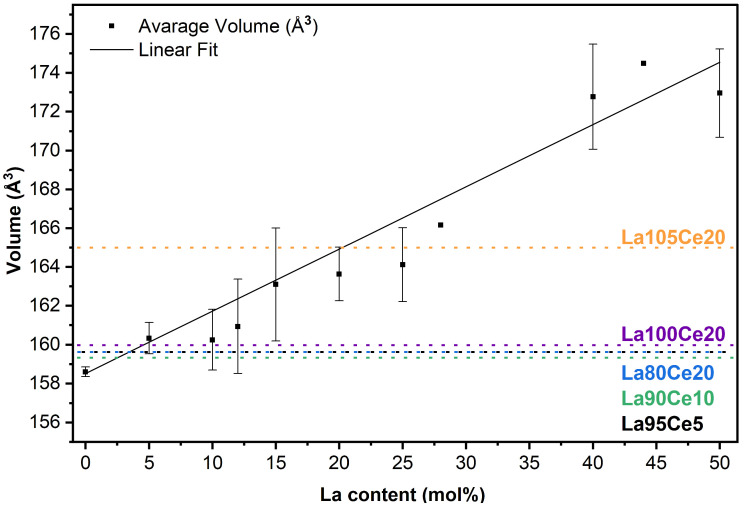
Calibration curve (solid line) of the average unit cell volume (black squares) versus La content for La-doped CeO_2_ (0 ≤ La mol% ≤ 50). The curve was constructed using structural data from the ICDD database [35], considering only high-quality PDF entries collected under ambient conditions. The variability in cell volume is expressed as standard deviation (black bars). Dotted colored lines indicate the fluorite unit cell volumes determined by the Rietveld method for La95Ce5, La90Ce10, La80Ce20, La100Ce20 and La105Ce20 compositions according to Table 2.

The volume of the fluorite-type CeO_2_ phase is also affected in all the investigated samples. Indeed, all cell volumes are significantly larger than the undoped ceria average volume of 158.60 Å^3^, calculated from structural data available in the ICDD database [35], suggesting the formation of the targeted lanthanum-doped ceria phase, since La^3+^ has a larger i.r. (1.36 Å in 12th-fold) than Ce^4+^ (1.14 Å in 12th-fold) [39]. To corroborate this finding, a calibration line of average cell volumes as a function of La content is depicted in Figure A2 for a series of La doped-CeO_2_ (0 ≤ La mol% ≤ 50). The curve was calculated on the basis of structural data listed in the ICDD database, selecting the most reliable PDF entries—high quality marks and ambient environment—and considering the cell volume statistical variability as the standard deviation [35]. Cross-referencing the calibration plot (Figure A2) with the Rietveld results (Table 2), it emerges that the La95Ce5, La90Ce10 and La80Ce20 samples exhibit a cell volume slightly larger than the undoped CeO_2_, suggesting a La mol % not higher than 5 mol%, whereas the cell volume of La100Ce20 and La105Ce20 falls markedly above that of the undoped CeO_2_ with the La content likely lying in the range of 5–10 mol% and below 25 mol%, respectively. The presence of La in the ceria phase can be attributed to the limited solubility of Ce in the perovskite lattice, which leads to an excess of La that is incorporated into CeO_2_ to maintain the overall A-site/B-site cation balance. The extent of La doping in ceria varies depending on the nominal composition, being higher in samples with greater relative La excess.

Looking at the microstructural data in Table A1, the crystal size of both the perovskite and fluorite phases in La105Ce20 and La100Ce20 samples is significantly smaller than that observed in the La95Ce5, La90Ce10 and La80Ce20 samples. This indicates that the two samples with greater relative La content are more defective, as a smaller crystal size is inherently associated with a higher degree of structural disorder in nanostructured materials. Consistently, in this sample the ceria unit cell volume is larger than in the other samples, indicating a higher incorporation of lanthanum into the ceria lattice, which leads to lattice expansion and increased structural distortion. Overall, these results indicate that the La105Ce20 sample exhibits the highest degree of defectivity, affecting both the perovskite and fluorite phases, and thus represents the most structurally disordered system among those investigated.

In summary, the low solubility of cerium promotes the formation of La-doped CeO_2_ during synthesis while simultaneously favoring the development of a defective, Fe^2+^/Co^2+-^ rich perovskite-fluorite composite via a one-pot route. On the other hand, lanthanum remains predominantly in the perovskite phase but is also partially incorporated into the ceria phase, forming a ceria–lanthana solid solution. This redistribution is required to maintain overall stoichiometry, as a significant fraction of cerium does not enter the perovskite, and LaFeO_3_ cannot tolerate either cation deficiency or excess [44]. As a result, an interplay of Ce and La between the perovskite and fluorite phases is established.

**Figure 4:**
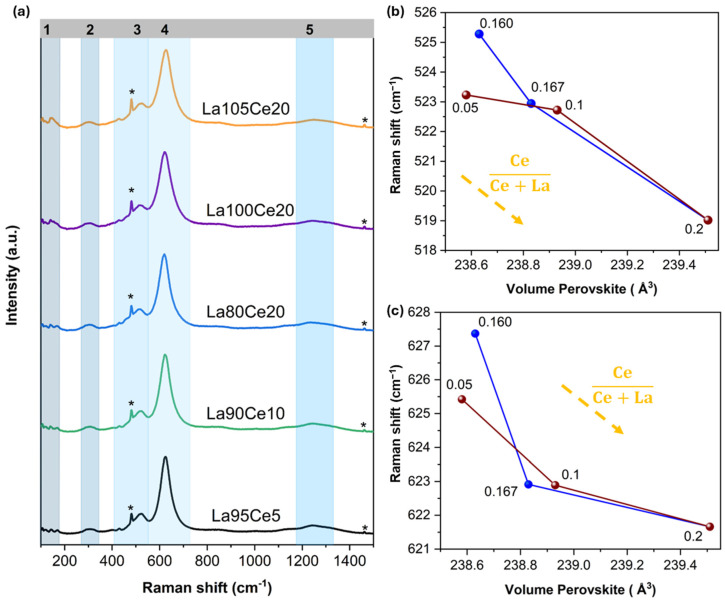
The Raman spectra of the perovskite-fluorite composites (**a**) and perovskite unit cell volume as a function of Raman shift at peak maximum in regions 3 and 4 (**b**,**c**). The yellow arrow indicates the trend of the calculated Ce nominal content; the red line refers to La95Ce5, La90Ce10 and La80Ce20 samples, and the blue line refers to La105Ce20, La100Ce20 and La80Ce20 (* signals arising from instrument chamber).

MicroRaman spectroscopy was employed to evaluate material structural distortion and defectivity through the investigation of the Raman active vibrational modes correlated to the Pbnm orthorhombic (24Γ = 7Ag + 7B1g + 5B2g + 5B3g) and Fm-3m cubic structures (F_2g_) [45,46]. Figure 4a shows the characteristic vibrational fingerprint of the orthorhombic perovskite structure, consistent with LaFeO_3_-type frameworks governed by four non-equivalent lattice positions (La, Fe, O1, O2). Instead, ceria-related bands are not clearly resolved, as masked by the dominant vibrational modes of the perovskite phase [45]. This observation is consistent with the predominance of the perovskite phase as revealed by the Rietveld refinement (Table 2), together with the limited probing volume of each Raman measurement (~1 µm). Moreover, peak broadening indicates an increased level of structural disorder, in agreement with the lattice expansion observed by XRD (Table 2). As displayed in Figure 4a, five distinct Raman shift regions are identified, corresponding to different types of local distortion The Raman-active modes are located at approximately 100–180 cm^−1^ (region 1), 260–350 cm^−1^ (region 2), 410–550 cm^−1^ (region 3), 550–720 cm^−1^ (region 4) and 1170–1350 cm^−1^ (region 5) [47,48,49]. According to the literature, region 1 is attributed to A-site cation displacement arising from La(Ce)-O vibration, while regions 2–4 correspond to BO_6_ rotation and tilting as well as to stretching and bending motions in O1-B-O2/B-O1/B-O2. The broad feature in region 5 is assigned to second-order Raman scattering, commonly reported for orthorhombic perovskites. The intense peak at ~622 cm^−1^ (region 4), along with the shoulder at ~500 cm^−1^, is generally attributed to B–O1/B–O2 stretching vibrations and is known to be highly sensitive to lattice defects such as oxygen vacancies and change in the B-site oxidation state in LaFeO_3_-based perovskites [41,46,47]. A more detailed view of this behavior is provided in Figure 4b,c, where the Raman shift at peak maximum is plotted as a function of the perovskite unit cell volume. A progressive shift towards low wavenumbers (red shift) is observed with an increase in the perovskite unit cell volume according to a more defective system, due to the partial reduction of B-site cations and oxygen vacancies. As previously discussed, the presence of La-doped ceria contributes to the broad spectral feature observed in the 410–550 cm^−1^ region, arising from the overlap between the B_3_g modes of the perovskite lattice and the characteristic F_2_g vibration of the fluorite-type cerium oxide, generally located near 460–465 cm^−1^ [20]. However, a distinct peak at ~465 cm^−1^ in this region is not clearly resolved. The observed broadening within the 410–550 cm^−1^ (region 3) window may instead reflect nanoscale dispersion of the ceria-based phase, spectral convolution with perovskite modes, or enhanced structural disorder. The Raman data indicate retention of long-range perovskite symmetry while revealing Ce-driven local structural perturbation and defect-sensitive evolution of the B–O framework.

**Figure 5:**
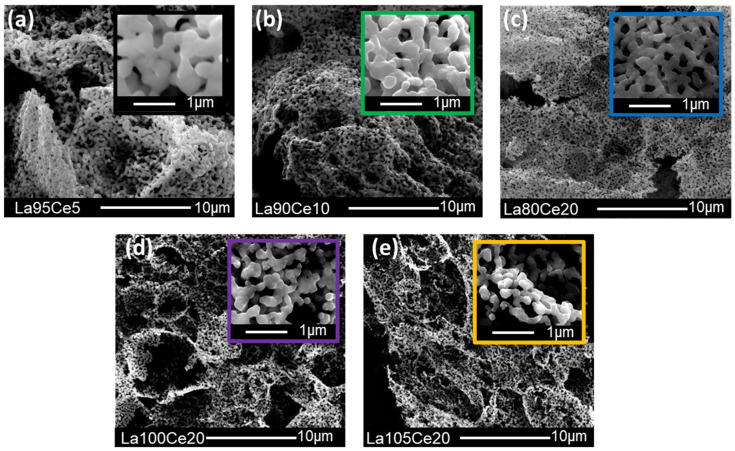
SEM images of the powder perovskite–fluorite composites La95Ce5 (**a**), La90Ce10 (**b**), La80Ce20 (**c**), La100Ce20 (**d**), and La105Ce20 (**e**). The main images are shown with 10 µm scale bars, while the insets provide higher-magnification details with 1 µm scale bars.

The powders’ morphology was evaluated by SEM analysis, as depicted in Figure 5. All calcined samples appear as agglomerates of nanoparticles with porous characteristics typical of materials prepared by solution combustion synthesis. It is well known that the relatively higher volume of gases released during the combustion event (Figure 1, step 3) leads to the formation of porous powders. These porous features remain recognizable even after high-temperature heat treatment, indicating that the microstructural templating effect of the fuel (citric acid), which burns during the combustion process, is capable of imprinting the powder morphology [37]. However, looking at Figure 5 and Table 3, some evident differences in the mean particle size range of the five samples that are visible seem to be related to the nominal Ce/(La + Ce) ratio. In particular, a narrower particle size range with smaller average dimensions is shown by samples having a higher ratio [40]. Despite powders exhibiting a multiphase nature, the perovskite phase is predominant (Table 2), and most of the particles observed by SEM might be reasonably attributed to the perovskite phase. These microstructural differences further support the hypothesis of a microstructural templating effect of the pre-formed ceria phase on the growth of the perovskite crystals during the one-pot synthesis.

The energy dispersive X-ray spectroscopy results (Table 3) reveal that the percentages of the metals are close to their nominal values, although in the probed EDX zone a slight lanthanum depauperation and a slight iron excess are observed.

**Figure 6:**
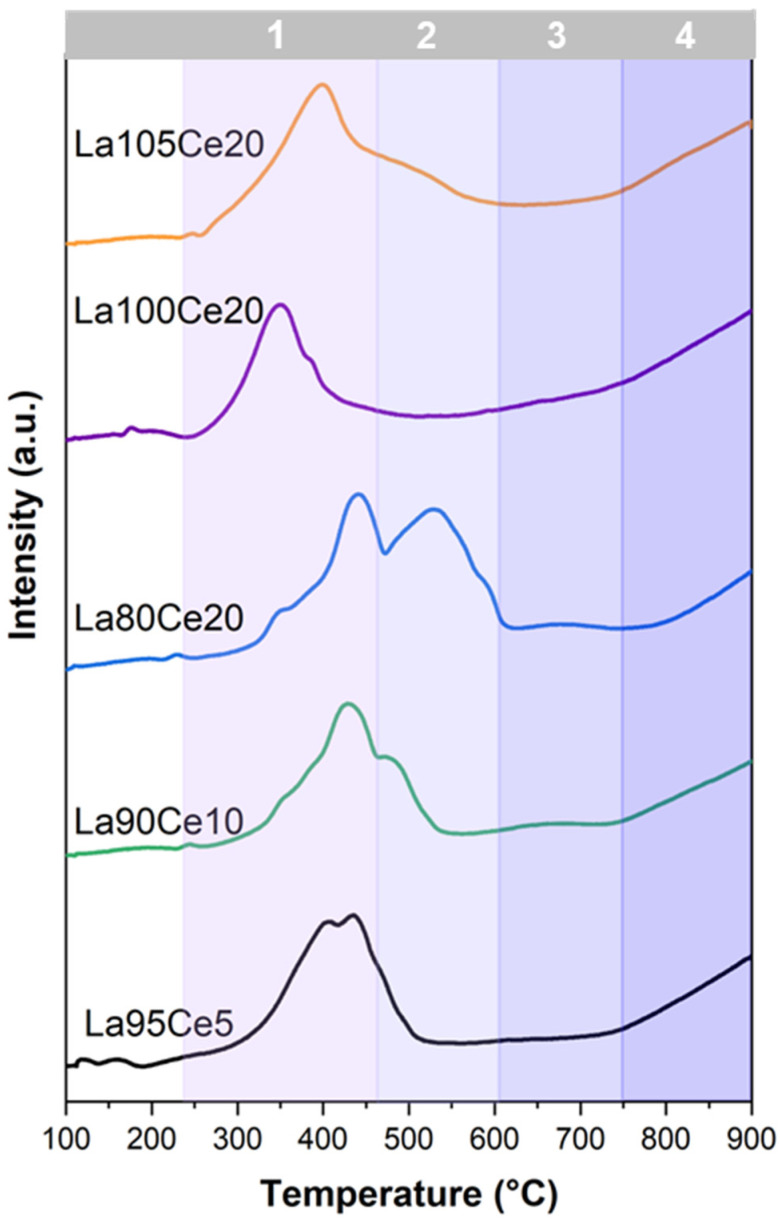
H_2_-TPR profiles of the perovskite-fluorite composites. The colored areas highlight the main reduction steps discussed in the text.

The reduction behavior of the perovskite-fluorite composites series was investigated through H_2_ temperature-programmed reduction (Figure 6). All samples exhibit hydrogen consumption over a broad temperature range, indicating multiple reduction processes involving B-site cations (Fe^3+^ and Co^3+^) as well as contributions from doped-ceria and iron oxide phases identified by the structural analysis (Table 2). The reduction profiles can be divided into four distinct ranges of temperature: a low-temperature region below ~450 °C (region 1), two intermediate regions in the range of ~450–600 °C (region 2) and ~600–750 °C (region 3), and a high-temperature region above 750 °C (region 4). The attribution of these reduction peaks agrees with the literature reports on analogous perovskite systems. In LaFeO_3_, the reduction proceeds stepwise, with Fe^3+^ → Fe^2+^ in the 430–550 °C range and subsequently Fe^2+^ to metallic Fe^0^ at temperatures above 700 °C. These processes are typically associated with low hydrogen consumption due to the strong stabilization of iron within the perovskite lattice [47,50,51]. Moreover, the literature reports attribute the low-temperature reduction feature (~300–500 °C) to the removal of surface-adsorbed oxygen species on LaFeO_3_. In contrast, LaCoO_3_ exhibits higher reducibility, characterized by two well-defined reduction steps: Co^3+^ → Co^2+^ (200–400 °C) and Co^2+^ → Co^0^ (450–650 °C) [52]. In the Co-doped LaFeO_3_ perovskites, these processes overlap, resulting in broadened hydrogen-consumption features that reflect the concurrent reduction of Fe and Co species [47]. Accordingly, the low-temperature region in the investigated perovskite-fluorite composites can be attributed to the reduction of Co^3+^ → Co^2+^, together with minor contributions from the removal of surface-adsorbed oxygen species (Figure 6, region 1). The intermediate regions (~450–700 °C) arise from overlapping reduction processes, including Co^2+^ → Co^0^ and Fe^3+^ → Fe^2+^ (Figure 6, regions 2,3). In all samples, the presence of La-doped CeO_2_ suggests that the surface Ce^4+^ → Ce^3+^ reduction may also contribute within this temperature interval. However, its individual contribution cannot be clearly distinguished due to overlap with the reduction of the transition-metal species [53]. At higher temperatures (>750 °C), hydrogen consumption is mainly associated with the reduction processes of Fe^2+^ → Fe^0^ and bulk ceria (Figure 6, region 4). In addition to the perovskite and ceria phases, the presence of segregated Fe_3_O_4_ contributes to the observed reduction behavior. According to the literature, magnetite undergoes stepwise reduction via Fe_3_O_4_ → FeO at intermediate temperatures (~300–500 °C), followed by FeO → Fe^0^ at higher temperatures (600–800 °C), which overlap with the reduction of the perovskite metal cations [54]. Consequently, both intermediate- and high-temperature regions likely include contributions from segregated iron oxide phases. This effect is evident in the 600–750 °C range for La95Ce5, La90Ce10 and La80Ce20, where the Fe_3_O_4_ content increases from 1.2 to 5.4 wt.% (Table 2), resulting in progressively pronounced high-temperature hydrogen consumption (Figure 6, region 3). Within the same La95Ce5, La90Ce10 and La80Ce20 series, a gradual increase in nominal cerium content further modifies the reduction profile. As the Ce loading increases across these compositions, additional intermediate temperature reduction peaks emerge. At higher nominal Ce content, the CeO_2_ phase gives rise to a characteristic reduction peak in the 500–600 °C range, in agreement with our previous findings (Figure 6, region 2) [53]. This contribution is particularly evident in La80Ce20, where the La-doped ceria exceeds 14 wt.% according to Rietveld refinement. Further insight can be gained by comparing La80Ce20 with La100Ce20 and La105Ce20, which contain similar amounts of CeO_2_-based phase but do not exhibit detectable iron oxide phase (Table 2). In La80Ce20, the first reduction peak shifts toward higher temperatures relative to La100Ce20 and La105Ce20, which can be attributed to the presence of segregated iron oxide species. This behavior may suggest that the iron oxide phase influences the reduction pathway, possibly influencing the oxygen mobility. In contrast, La100Ce20 exhibits a reduction peak at lower temperature, indicating enhanced reducibility relative to the other compositions. This peak can be associated with the sequential reduction of Co^3+^ → Co^2+^ → Co^0^ strongly interacting with the CeO_2_-based phase, with possible additional contribution from surface ceria reduction in the same temperature range. In contrast, La105Ce20 presents a higher contribution from CeO_2_ surface reduction, highlighting the different weight percentages of the perovskite and ceria phases and, consequently, suggesting different interactions of the two phases. These results demonstrate that variations in the nominal Ce content not only lead to different phase compositions but also influence composite reducibility. Overall, TPR has proven to be an effective technique to compare reduction behavior and phase interactions in perovskite-fluorite composites. The observed shifts of reduction peaks toward low temperatures indicate enhanced oxygen mobility, which could be beneficial for oxygen exchange and transport in SOFC cathode materials.

**Figure 7:**
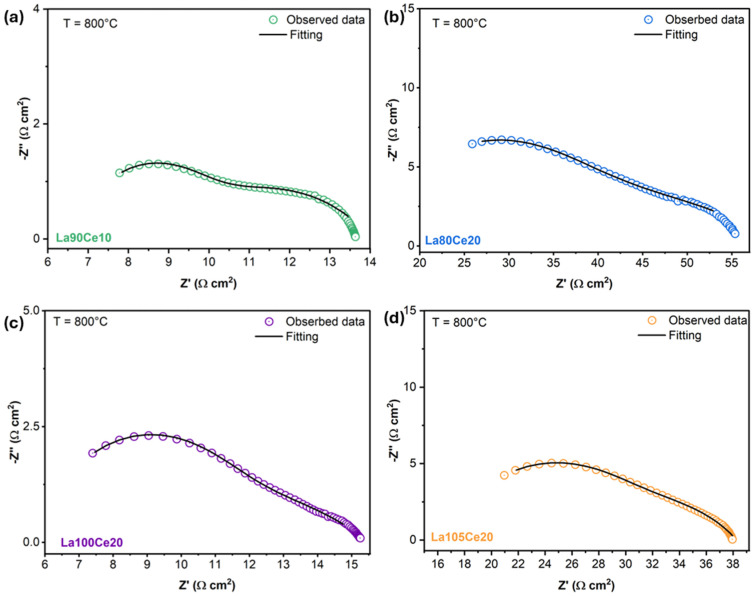
Nyquist plots (colored circles) and equivalent circuit fittings (black line) of La90Ce10 (**a**), La80Ce20 (**b**), La100Ce20 (**c**) and La105Ce20 (**d**).

**Figure A3:**
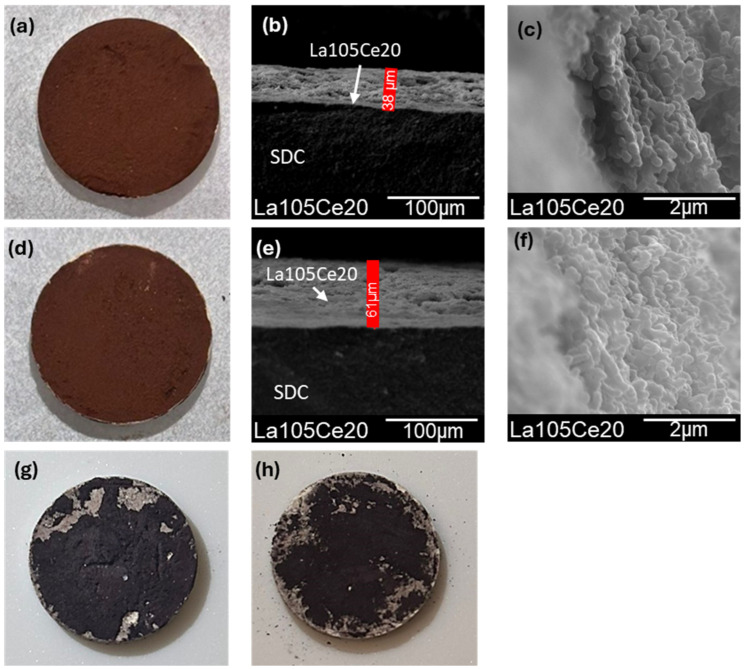
La105Ce20/SDC/La105Ce20 MEA as a representative example: pre- and post-test pictures (**a**–**d**), pre- and post-test SEM cross-section images (**b**–**e**), pre- and post-test SEM electrode images (**c**–**f**). Both sides of the La95Ce5/SDC/La95Ce5 MEA (**g**,**h**).

Electrochemical impedance spectroscopy was performed to evaluate the investigated composites as cathode materials for the ORR. All compositions were successfully deposited on SDC, except La95Ce5 (Figure A3a,d,g,h). As shown in Figure A3, the comparison between the pre- and post-test photographs and SEM micrographs of the La105Ce20/SDC/La105Ce20 MEA, taken as a representative sample, reveals that the membrane preserves its overall integrity after testing. In particular, the cross-sectional and electrode-surface SEM images show good adhesion between the electrode and electrolyte layers, without evident delamination or severe microstructural degradation. Figure 7 depicts the Nyquist plots acquired for all tested symmetrical cells at 800 °C, with the relative equivalent circuit fits.

**Figure 8:**
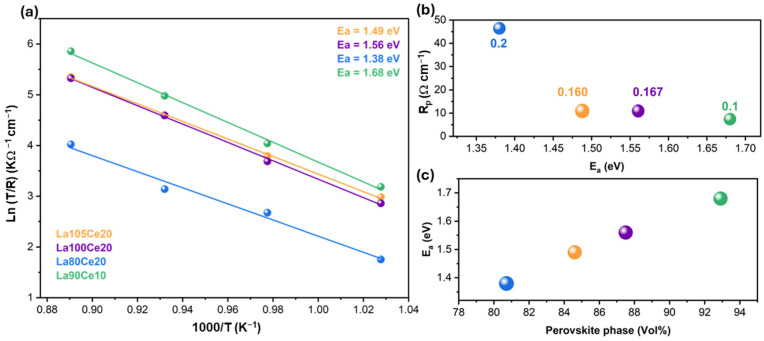
Arrhenius plots (**a**), polarization resistance at 800 °C as a function of the activation energy (with indication of calculated nominal Ce content) (**b**), and activation energy as a function of volume fraction of perovskite phase (**c**) of the investigated samples. Volume fractions were calculated from weight fractions using theoretical densities obtained from Rietveld refinements.

The impedance response of these systems was modeled using an equivalent circuit composed of one resistance (R_s_) and two RQ elements connected in series—R_s_(R_1_Q_1_)(R_2_Q_2_)—where R represents the resistance and Q the circuital constant phase element (CPE). The capacitance associated with the CPE can be expressed as $C=(R^{1-n}Q{)}^{1/n}$, where C is the capacitance and *n* is an additional fitting parameter [55]. The high-frequency contribution associated with the electrolyte is represented by R_s_, while the two RQ elements describe the intermediate-frequency response related to processes at the electrode/electrolyte interface and the low-frequency contribution attributed to electrode transport processes, mainly governed by surface oxygen exchange and bulk charge storage. The total electrode resistance is given by the sum of R_1_ and R_2_ and is generally referred to as the polarization resistance, R_p_. The Arrhenius plots in Figure 8a, obtained from the temperature dependence of R_p_, provide insight into the activation energy of the electrode processes associated with oxygen reduction and oxygen transport. The calculated activation energies, E_a_, follow the order La80Ce20 (1.38 eV) < La105Ce20 (1.49 eV) < La100Ce20 (1.56 eV) < La90Ce10 (1.68 eV). Although La80Ce20 exhibits the lowest activation energy, it shows the highest polarization resistance. Conversely, La90Ce10 displays lower polarization resistance despite its higher activation energy. According to the Arrhenius law, in mixed ionic-electronic conductors, both positive and negative carriers contribute to the overall conductivity. Consequently, the *R_p_*, expressed also as area-specific resistance (ASR, i.e., normalized to the electrode area), is strongly influenced by both the concentration and mobility of oxygen vacancies and electrons in the cathode. Focusing on *V_Ö_* contribution to positive charge transport, the lower ASR observed for La90Ce10 suggests an increase in the product of vacancy concentration and mobility, since more mobile vacancies facilitate oxygen ion transport and surface exchange. However, the activation energy derived from the Arrhenius plots reflects the combined contributions of oxygen vacancy-mediated transport and interfacial processes. The higher E_a_ observed for La90Ce10 compared to La80Ce20 and the other samples is indicative of less efficient charge transport and slower oxygen reduction processes. The high resistance observed for La80Ce20 may instead arise from an excessive concentration of vacancies as well as the higher volume of grain boundaries resulting from narrower particle size distribution. At high vacancy concentrations, vacancy–vacancy interactions may occur, leading to vacancy clustering or local lattice distortions, which ultimately hinder oxygen transport and surface exchange processes. This phenomenon could be amplified when there are a greater number of grain boundaries. Focusing on the other two compositions, the Arrhenius plots reveal that the La105Ce20 and La100Ce20 exhibit comparable behavior. In addition, the *R_p_–E_a_* correlation indicates that these two compositions provide the best compromise between polarization resistance and activation energy compared with La90Ce10 and La80Ce20 (Figure 8b). As shown above, Rietveld analysis indicates the coexistence of two phases for these two compositions (Table 2), suggesting that interaction between the perovskite and fluorite phases may provide an optimal balance, possibly promoting oxygen transport and surface exchange processes. However, the La105Ce20 sample, with a nominal Ce content of 0.16, appears to represent the best composition, for maximizing the amount of ceria-based phase while maintaining the highest La-loading, as reflected by its unit cell volume. Considering the predominance of the perovskite phase in all composites, its relative fraction is expected to critically influence the electrochemical response. As shown in Figure 8c, the activation energy exhibits an approximately linear dependence on the perovskite phase, as the volume fraction, increasing with the perovskite content. This trend further explains the lowest E_a_ observed for La80Ce20, which contains the lowest fraction of perovskite, while La90Ce10, with the highest perovskite content, exhibits the highest activation energy. This linear relationship should suggest that the presence of La-doped ceria contributes to lowering the activation energy of the electrode processes, indicating that the ceria phase plays a beneficial role in facilitating oxygen-related transport processes. However, La80Ce20 and La90Ce10 also contain a fraction of magnetite. The presence of this additional phase may influence the electrochemical behavior, and therefore the observed trend cannot be attributed solely to the relative amount of ceria phase.

In contrast, the polarization resistance does not follow a strictly linear dependence on the perovskite fraction. Although the perovskite phase represents the electrocatalytic active component, and therefore a higher perovskite content would be expected to reduce R_p_, the observed trend deviates from simple proportionality. This behavior indicates that electrochemical performance is not governed solely by the amount of active perovskite phase but mainly by the interactions between the perovskite and ceria-based phases. As discussed above, the biphasic systems represent the best composites. La105Ce20 and La100Ce20 exhibit lower R_p_ values than expected from a simple compositional trend, highlighting the beneficial role of the perovskite-fluorite interaction in promoting oxygen transport and surface exchange processes.

## 4. Conclusions

Sr-free perovskite-fluorite composites based on LaFe_0.8_Co_0.2_O_3_ were successfully synthesized by a one-pot solution combustion synthesis to promote the formation of a perovskite phase together with a limited amount of a fluorite-type ceria phase. Structural analysis indicates that the incorporation of Ce at the A-site of the perovskite occurs only to a small extent, while the majority forms fluorite-type La-doped ceria. Despite its limited incorporation into the perovskite lattice, the overall cerium content significantly affects the defect chemistry of the perovskite. In particular, the progressive increase in the perovskite unit cell volume with increasing nominal Ce content suggests modifications in the oxidation state of B-site cations consistent with expansion of the BO_6_ octahedra. MicroRaman spectroscopy further supports this finding, revealing changes in the vibrational features associated with the perovskite framework and indicating defect-induced distortions of the BO_6_ octahedra. This defectivity is ascribed to an interaction of perovskite precursors with the doped ceria phase, which acted during the one-pot synthesis as a structural template for the crystal growth of the perovskite phase. This hypothesis is further supported by the SEM results, which show a clear progressive decrease in the average particle size with increasing nominal cerium content. In all samples, the lanthanum is accommodated in both perovskite and fluorite structures, causing ceria cell volume expansion and increasing defectivity. The interplay of La and Ce between the two phases promotes the formation of composites with proper charge transport and redox properties. Overall, among the investigated composites, La105Ce20 exhibits the most favorable electrochemical performance, providing the best compromise between polarization resistance and activation energy. In conclusion, the one-pot solution combustion synthesis route represents an effective strategy to promote favorable interactions between perovskite and fluorite phases, enabling the development of Sr-free perovskite-fluorite composites with reduced ceria content and enhanced functional properties. The combined use of X-ray diffraction, microRaman spectroscopy, and temperature-programmed reduction analysis proves to be a powerful and versatile approach for elucidating cation interplay between phases, defect chemistry, and redox behavior in these systems. Overall, these findings provide valuable insights that can guide the rational design of novel perovskite oxide–based composites for advanced catalytic and electrocatalytic applications. In this regard, in situ/operando approaches, chemical compatibility electrolyte/electrodes and extended stability tests may offer useful insights for optimizing the design of these composites.

## Figures and Tables

**Table 1:** List of the prepared samples.

Label	Nominal Composition	Nominal Ce Content Ce/(La + Ce)
La105Ce20	La_1.05_Ce_0.2_Fe_0.8_Co_0.2_O_3_	0.160
La100Ce20	LaCe_0.2_Fe_0.8_Co_0.2_O_3_	0.167
La80Ce20	La_0.8_Ce_0.2_Fe_0.8_Co_0.2_O_3_	0.200
La90Ce10	La_0.9_Ce_0.1_Fe_0.8_Co_0.2_O_3_	0.100
La95Ce5	La_0.95_Ce_0.05_Fe_0.8_Co_0.2_O_3_	0.050

**Table 2:** Rietveld refinement results for the investigated samples.

Label	Perovskite Phase (wt%)	CeO_2_ Phase (wt%)	Fe_3_O_4_ Phase (wt%)	Perovskite Phase Volume (Å^3^)	CeO_2_ Phase Volume (Å^3^)	Reliability Factors ^a^ (%)
La105Ce20	84.4 ± 0.2	15.6 ± 0.2	0	238.63 ± 0.02	165.03 ± 0.05	wR = 5.29 GOF = 3.31
La100Ce20	87.2 ± 0.2	12.8 ± 0.2	0	238.83 ± 0.02	160.12 ± 0.03	wR = 4.69 GOF = 2.9
La80Ce20	80.1 ± 0.1	14.2 ± 0.1	5.7 ± 0.1	239.509 ± 0.003	159.49 ± 0.01	wR = 3.22 GOF = 2.14
La90Ce10	92.7 ± 0.2	5.4 ± 0.1	1.9 ± 0.1	238.93 ± 0.02	159.39 ± 0.02	wR = 5.98 GOF = 5.70
La95Ce5	96.0 ± 0.1	2.83 ± 0.01	1.22 ± 0.01	238.576 ± 0.004	159.48 ± 0.06	wR = 4.90 GOF = 3.14

^a^ wR: the weighted-profile R-factor; GOF: goodness-of-fit [38].

**Table 3:** Range of particle sizes, nominal metal atomic composition, and EDX metal atomic composition.

Label	Range of Particle Size (nm)	Nominal Metal Atomic %				EDX Metal Atomic %			
		La	Ce	Fe	Co	La	Ce	Fe	Co
La105Ce20	100–200	46.6	8.9	35.5	8.9	41.0 ± 0.8	8.0 ± 0.2	42.0 ± 0.8	9.0 ± 0.2
La100Ce20	100–200	45.5	9.1	36.4	9.1	41.5 ± 0.8	7.5 ± 0.2	43.0 ± 0.8	8.0 ± 0.2
La80Ce20	80–180	40	10	40	10	39.0 ± 0.8	9.5 ± 0.2	41.0 ± 0.8	10.5 ± 0.2
La90Ce10	150–400	45	5	40	10	41.0 ± 0.8	4.5 ± 0.1	43.5 ± 0.9	11.0 ± 0.2
La95Ce5	200–400	47.5	2.5	40	10	44.0 ± 0.8	2.7 ± 0.1	43.0 ± 0.9	10.3 ± 0.2

## Data Availability

The original contributions presented in this study are included in the article. Further inquiries can be directed to the corresponding author.

## Appendix A

**Table A1:** Crystal size values with uncertainties of the perovskite and fluorite phases, as obtained from Rietveld refinement of the X-ray diffraction data for the investigated samples.

Label	Perovskite Phase Crystal Size (nm)	CeO_2_ Phase Crystal Size(nm)
La105Ce20	84 ± 1	38 ± 1
La100Ce20	77 ± 1	48 ± 1
La80Ce20	337 ± 6	112 ± 2
La90Ce10	252 ± 9	116 ± 10
La95Ce5	267 ± 6	104 ± 8
